# Closing Sexual Health Service Gaps With a New Service Model in Germany: Performance of an on-Site Integrated, Cross-Sectoral, Low Threshold Sexually Transmitted Infections/HIV Counseling and Treatment Service

**DOI:** 10.3389/fpubh.2022.793609

**Published:** 2022-04-25

**Authors:** Matthias C. Müller, Susanne Usadel, Stefan Zimmermann, Andreas Fahrhöfer, Winfried V. Kern, Ulrike Hoffmeister, Siegbert Rieg

**Affiliations:** ^1^Division of Infectious Diseases, Department of Medicine II, Faculty of Medicine, Medical Center, University of Freiburg, Freiburg im Breisgau, Germany; ^2^Department of Infection Medicine, Medical Service Centre Clotten, Freiburg im Breisgau, Germany; ^3^Checkpoint Aidshilfe Freiburg e.V., Freiburg im Breisgau, Germany

**Keywords:** HIV, STI, sexual health, prevention, public health, integrated, cross-sectoral

## Abstract

**Purpose:**

In Germany, the incidence of bacterial sexual transmitted infections (STI) is on the rise and still high for HIV infections. The Center for Sexual Health Freiburg (CSHF) was established to offer low threshold access for STI/HIV counseling, testing, HIV pre-exposure prophylaxis (PrEP), and on-site treatment. The objective of this study was to analyze the performance of CSHF.

**Methods:**

Longitudinal study that includes all clients presenting between 1 May 2020 and 28 February 2021 at CSHF and willing to sign informed consent.

**Results:**

In the study period, 536 clients presented at CSHF of whom 417 clients were included in the study resulting in 668 client contacts. Clients' median age was 28.1 years (range: 18.0–73.1), 55.9% were men, 42.0% were women, 0.3% were transman, and 1.7% were not binary. Clients' sexual orientation was heterosexual (56.6%), homosexual men (26.2%), and bisexual (13.6%). STI screening resulted in the detection of any STI in 3.4% (95% confidence interval (CI): 0.7–6.1) of women, in 3.1% (95% CI: 0.0–6.5) of heterosexual men, and in 22.2% (95% CI: 13.0–31.5) of men having sex with men (MSM) not taking PrEP. Eighty-one MSM received PrEP with a total follow-up of 57.3 person-years and 0.44 STIs per person-year.

**Conclusion:**

The substantial burden of STI in the study population emphasizes the need for regular and low threshold STI screening services. The concept of CSHF may facilitate access to STI/HIV counseling, testing, and PrEP for a wide spectrum of people and may prove to be an important contribution to the efforts to reduce STI and HIV incidence in Germany.

## Introduction

The incidence of bacterial sexual transmitted infections (STI) that include *Chlamydia trachomatis* (CT), *Neisseria gonorrhoeae* (NG), and syphilis is steadily rising in the European Union and the United States (1, 2). In Germany, the number of syphilis cases quadruplicated since 2001 and reached a new peak in 2019 with about 8,000 cases (3). The epidemiology of NG infections is less well characterized as it is not a notifiable disease in Germany. The evolution on the national level is probably comparable to temporal trends in the federal state of Sachsen, where gonorrhea is a notifiable disease, showing a 10-fold increase of rates from 1.8 infections per 100,000 population in 2001 to 19.9 per 100,000 population in 2019 (4). CT is the most common bacterial STI worldwide with a crude notification rate of 146 per 100,000 population for the 23 European Union (EU)/European Economic Area (EEA) countries in 2018 (5). Data on the situation in Germany are scarce, prevalence was 5.3% in women, 3.5% in men having sex with men (MSM), and 3.2% in heterosexual men attending an HIV-counseling service in Germany (6). The incidence of newly diagnosed HIV infections declined from about 3000 cases in the year 2016 to 2000 cases in the year 2020 - in Germany–an evolution that may be related to the introduction of pre-exposure prophylaxis (PrEP) (7).

A hallmark of many STIs is their asymptomatic nature, thus a significant proportion of STIs remains undetected and serves as a reservoir for further transmission. Moreover, untreated STI may eventually result in serious long-term sequelae (8–12). In Germany in 2018, around 12% of people were estimated to be unaware of their HIV infection and one-third of individuals had CD4 counts below 200 cells/μl or AIDS at the time of HIV diagnosis (7). To tackle the problem of asymptomatic infections, medical guidelines recommend STI testing for asymptomatic persons at risk (13).

Access to STI/HIV consulting and testing, timely provision of adequate therapy, and delivering vaccination to protect against hepatitis A virus (HAV) and hepatitis B virus (HBV) are cornerstones in national and international HIV/STI-prevention strategies (14, 15). In Germany, costs for STI screening are only covered by compulsory health insurance for persons taking HIV PrEP and for CT diagnostic in women younger than 25 years. Considering the high costs of comprehensive STI screening, which easily exceeds 200€, accessibility to STI testing is restricted for many asymptomatic individuals. Affordable STI testing services are only available at local public health authorities and non-governmental organizations. Both institutions have in common that they are not authorized to prescribe medication, thus they can neither provide STI treatment or vaccination against HAV and HBV nor PrEP.

To tackle this shortfall and improve accessibility for sexual health counseling, the Center for Sexual Health Freiburg (CSHF) was conceived as an integrated, cross-sectoral, low threshold service-providing anonymous STI/HIV counseling, diagnostics, treatment, and PrEP at one site. CSHF is a part of emerging development of intersectoral sexual health centers (ISHC) in Germany reacting to gaps in sexual healthcare provision in the national healthcare system. To our best knowledge, three other ISHCs were independently established in the German metropoles of Bochum (WIR–Walk IN Ruhr, Center for Sexual Health and Medicine), Berlin (Checkpoint BLN), and Frankfurt am Main (Checkpoint Frankfurt) since 2016 (16–18). The situation of CSHF in Freiburg, the regional center of a more rural catchment area in south-west Germany, may have implications for the composition of the population, the sexual behavior, the epidemiology of STI, and the management of clients and patients.

The aim of this study was to describe the utilization of the CSHF, to elucidate the sociodemographic characteristics, and affiliation to risk groups of clients attending the CSHF and to assess the prevalence of STI in this population.

## Materials and Methods

### Ethics Consideration

The study was approved by the institutional ethics review board of the medical center of the University of Freiburg (no.: 78/20, 18.02.2020) and was registered in the German Clinical Trials Register (DRKS00020957). All patients included in this study provided written informed consent.

### Study Setting/Design

This study is a prospective cohort study that included all clients presenting between 1 May 2020 and 28 February 2021 at CSHF willing to participate in this study and provide written informed consent. The service provided STI/HIV counseling and testing for asymptomatic clients. CSHF was conceived as a walk-in service during opening hours in the late afternoons on 2 weekdays. To avoid crowding during the severe acute respiratory syndrome coronavirus 2 (SARS-CoV-2) pandemic, CSHF worked with appointment-based consultations during the study period. CSHF is in Freiburg, a regional center and University City in the south-west of the federal state of Baden-Württemberg in Germany. The population of Freiburg and its catchment area are about 220,000 and 660,000, respectively (19).

All clients presented at CSHF were asked to fill out an anonymous questionnaire that was provided by the German Public Health Institute (Robert Koch Institute–RKI) to community-based STI/HIV test and counseling facilities in Germany addressing socio-economic aspects, sexual orientation, behavior, and health. Subsequently, all clients received sexual health counseling. Standardized testing for STI according to standard operating procedures (following national STI guidelines) was offered irrespective of perceived risk that includes off-site laboratory-based serology for HIV, HBV, syphilis, and self-collected pooled nucleic acid amplification tests (NAAT) for CT and NG from three localizations (men: rectal and pharyngeal swabs, and urine sample; women: rectal, pharyngeal, and vaginal swabs). Serology for HAV and HCV was proposed to MSM only. The complete visit that included counseling and testing was charged EUR 10.00. Clients were informed by phone about the results of their tests. If therapeutic medical interventions were indicated, clients were offered to present at the on-site medical practice.

HIV PrEP and related testing, which is covered by compulsory health insurance since September 2019, were also provided on-site in accordance with national PrEP guidelines (20). PrEP services included counseling, monitoring for potential adverse reaction, vaccination against HAV and HBV, prescription of PrEP, and STI screening that includes off-site laboratory-based serology for HIV, HBV, HCV, syphilis, and pooled NAAT for CT and NG from rectal, pharyngeal, and urethral swabs. PrEP users with asymptomatic or symptomatic STI were offered to receive treatment on-site.

### Statistical Analysis

Continuous variables were summarized with medians, range, interquartile ranges (IQRs), and categorical variables with frequencies and percentages. Differences were analyzed using the chi-squared test and the Wilcoxon-Mann-Whitney test as appropriate. Person-years of follow-up of PrEP users were calculated from the beginning to the end of the study period for prevalent clients and from the date of the first presentation to the end of the study period for incident clients. Incidence per person-years was calculated for STI. Data were analyzed using STATA version 12.1 (StataCorp; College Station, TX, USA).

Spatial distribution of place of residence of clients was visualized using Epi Info™, CDC, Atlanta, GA, USA. Localization on the map corresponds to the address of a public institution with the postcode of the respective client.

## Results

**Figure 1:**
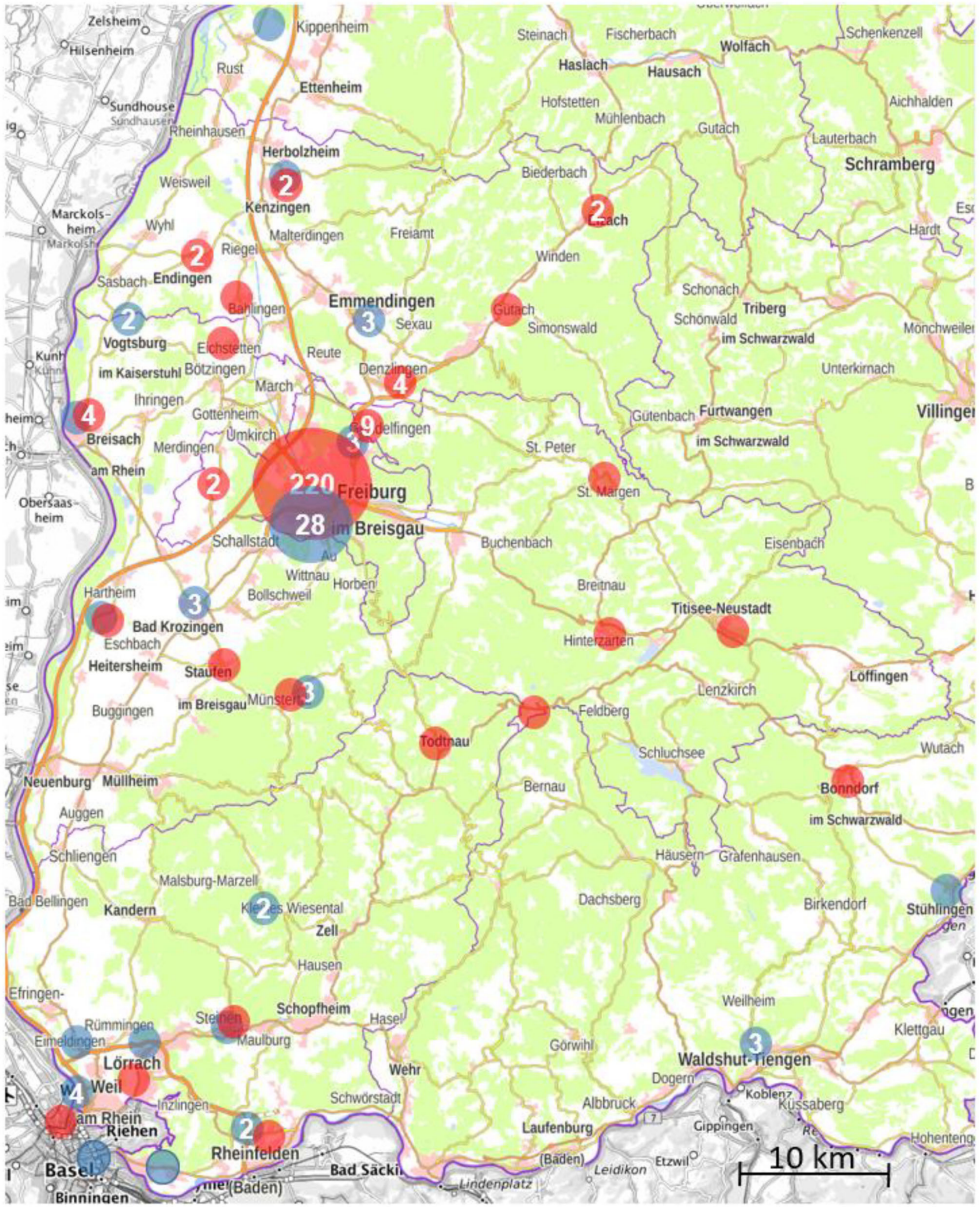
Places of residence of clients according to postcode presenting at the Center for Sexual Health Freiburg, Germany, between 1 May 2020 and 28 February 2021. Red dots correspondent to clients presenting for sexual transmitted infection (STI)/HIV counseling, blue dots to pre-exposure prophylaxis (PrEP) users.

A total of 536 patients were presented at CSHF in the investigation period of whom 417 patients were willing to participate in the study and signed the informed consent. Due to capacity reasons, 69 requests for appointments could not be granted. The majority of clients presenting at CSHF lived in the district of Freiburg but clients came from all parts of the metropolitan area and beyond (Figure 1). Median age of clients was 28.1 years (range: 18.0–73.1) and two-thirds (*n* = 272, 66.0%) were younger than 30 years (Table 1). Clients described themselves as being men in 55.9%, women in 42.0%, transman in 0.3%, and as not binary in 1.7%. Sexual orientation of the clients was heterosexual in 56.6%, homosexual men in 26.2%, bisexual in 13.6%, and others in 3.6%. Taken together, 135 (32.4%) MSM of whom 81 (19.4%) were taking PrEP. The median number of presentations was 1 (IQR 1–3) for clients who presented for STI/HIV counseling and 4 (IQR 2-−7) for MSM using PrEP resulting in a total of 667 client contacts. Clients were predominantly from Germany (84.6%) and other European countries (7.2%), and most of them were insured for healthcare in Germany (96.9%).

**Table 1:** Baseline characteristics of clients presenting at the Center for Sexual Health Freiburg, Germany, between 1 May 2020 and 28 February 2021.

	**All**	**Female**	**Heterosex. men**	**MSM w/o PrEP**	**Other^a^**	**MSM with PrEP**
N (%)	417	175 (42.0)	98 (23.5)	54 (12.9)	9 (2.16)	81 (19.4)
Age, years-median (range)	28.1 (18.0-73.1)	25.5 (19.7-44.2)	28.5 (19.5-48.1)	28.0 (21.5-73.1)	22.9 (19.1-44.5)	35.0 (18.0-56.9)
18–30 years, n (%)	272 (66.0)	138 (79.3)	68 (70.1)	36 (67.9)	8 (88.9)	22 (27.9)
31–60 years, n (%)	137 (33.3)	36 (20.7)	29 (29.9)	14 (26.4)	1 (11.1)	57 (72.2)
> 60 years, n (%)	3 (0.7)	0	0	3 (5.7)	0	0
Place of birth n (%)						
Germany	352 (84.6)	153 (87.4)	84 (85.7)	44 (81.5)	8 (88.9)	63 (77.8)
Other Europe	30 (7.2)	12 (7.9)	6 (6.1)	4 (7.4)	1 (11.1)	7 (8.6)
Asia	12 (2.9)	3 (1.7)	2 (2.0)	3 (5.6)	0	4 (4.9)
South America	7 (1.7)	2 (1.1)	2 (2.0)	2 (3.7)	0	1 (1.2)
Africa	4 (1.0)	1 (0.6)	1 (1.0)	0	0	2 (2.4)
Other	11 (2.6)	4 (2.3)	3 (3.1)	1 (1.9)	0	4 (4.9)
Educational attainment n (%)						
University degree	181 (43.8)	64 (36.8)	57 (58.8)	18 (34.0)	2 (25.0)	40 (49.4)
In academic/ professional education	147 (35.6)	88 (50.6)	24 (24.7)	20 (37.7)	5 (62.5)	10 (12.4)
Professional degree	79 (19.3)	20 (11.5)	16 (16.5)	13 (24.5)	0	30 (37.0)
No academic/ professional degree	6 (1.5)	2 (1.2)	0	2 (3.8)	1 (12.5)	1 1 (0.2)
Health insurance in Germany n (%)	401 (96.9)	171 (98.3)	94 (96.9)	51 (96.2)	8 (88.9)	77 (95.1)

*Heterosex., heterosexual; MSM, men having sex with men; PrEP, HIV pre-exposure prophylaxis; w/o, without*.

a *Sex: Transman n = 1, not binary n = 7, other n = 1*.

Clients presenting for STI/HIV screening (*n* = 336) had received testing for NG in 88.8% (*n* = 295) and for CT in 86.9% (*n* = 292). Serology for syphilis was available in 86.0% (*n* = 289), for HIV in 89.9% (*n* = 302), for HBV in 83.3% (*n* = 280), and for HCV in 12.8% of clients.

Of all clients, 8.6% (95% confidence interval (CI): 5.9–11.3) had at least one STI over the entire study period (Table 2). Prevalence of any STI was similar in women and in heterosexual men (3.4% [95% CI: 0.7–6.1] vs. 3.1% [95% CI: 0.0–6.5], *p* = 0.87) and higher in MSM not taking PrEP (16.7%, 95% CI: 6.4–26.9) when compared to women (*p* < 0.01) and heterosexual men (*p* < 0.01). In women, heterosexual men, and MSM not taking PrEP, prevalence of CT was 2.9% (95% CI: 0.4–5.3), 3.1% (95% CI: 0.0–6.5), and 11.1% (95% CI: 2.5–19.8), and of NG 1.1% (95% CI: 0.0–2.7), 0%, and 1.9% (95% CI: 0.0–5.6), respectively.

**Table 2:** Sexual behavior and health of clients presenting at the Center for Sexual Health Freiburg, Germany, between 1 May 2020 and 28 February 2021.

	**All**	**Female**	**Heterosexual men**	**MSM without PrEP**	**Other^a^**	**MSM with PrEP**
Number of persons having had un–protected vaginal or anal intercourse with^b^ -*n* (%)						
Nobody	65 (17.8)	24 (15.5)	18 (22.2)	13 (28.9)	1 (14.3)	9 (12.9)
1 – 3	248 (67.8)	118 (76.1)	64 (71.9)	26 (57.8)	4 (57.1)	36 (51.4)
4 – 5	31 (8.5)	10 (6.5)	6 (6.7)	3 (6.7)	2 (28.6)	10 (14.3)
6 – 10	17 (4.6)	3 (1.9)	0	2 (4.4)	0	12 (17.1)
≥ 11	5 (1.4)	0	1 (1.1)	1 (2.2)	0	3 (4.3)
Clients with ≥ 1 STI -*n* (%, 95% CI)	36 (8.6; 5.9–11.3)	6 (3.4; 0.7–6.1)	3 (3.1, 0.0–6.5)	9 (16.7; 6.4–26.9)	0	18 (22.2, 13.0–31.5)^e^
STIs – *n* (%, 95% CI)	44	7	3	9	0	25^e^
Syphilis	9 (2.2, 0.8–3.6)	0	0	2 (3.7, 0.0–8.9)	0	7 (8.6, 2.3–14.9)^e^
*C. trachomatis*	24 (5.8, 3.5–8.0)	5 (2.9, 0.4–5.3)	3 (3.1, 0.0–6.5)	6 (11.1, 2.5–19.8)	0	10 (12.4, 5.0–19.7)^e^
*N. gonorrhoeae*	9 (2.2, 0.8–3.6)	2 (1.1, 0.0–2.7)	0	1 (1.9, 0.0–5.6)	0	6 (7.4, 1.6–13.2)^e^
LGV	1 (0.2, 0.0–0.7)	0	0	0	0	1 (1.2,0.0–3.7)^e^
HIV	1 (0.2, 0.0–0.7)	0	0	0	0	1 (1.2,0.0–3.7)^e^
HBV	0	0	0	0	0	0^e^
HCV	0	0	0	0	0	0^e^
Protective Anti–HAV–IgG–Titer^c^-*n* (%, 95% CI)	n.a.	n.a.	n.a.	25 (65.8, 50.0–81.6)	n.a.	74 (91.4, 83.0–96.5)^f^
Protective Anti–HBs–Titer^d^ *-n* (%, 95% CI)	114 (45.6, 39.4–51.8)	58 (46.8, 37.9–55.7)	24 (34.3, 22.9–45.7)	18 (47.4, 30.7–64.0)	8 (87.5, 57.9–100)	72 (88.9, 80.0–94.8)^f^

*LGV, lymphogranuloma venereum; HBV, hepatitis B virus; HCV, hepatitis C virus; HAV, hepatitis A virus; IgG, immunoglobulin G; MSM, men having sex with men; STI, sexual transmitted infection; PrEP, HIV pre–exposure prophylaxis; CI, confidence interval; n.a., not applicable*.

a *Sex: Transman n = 1, not binary n = 7, other n = 1;*

b *6 months prior to first presentation;*

c *Anti–HAV–IgG–Titer > 100 IE/l;*

d *Anti–HBs–Titer ≥ 100 IE/l;*

e *cumulative incidence;*

f *at the end of the study period*.

The two women who tested positive for NG were 20 and 23 years old and indicated unprotected sexual intercourse with 0 and 4–5 partners in the last 6 months prior to presentation. Women with CT infection were in median 30.4 years old (IQR: 29.7–34.9) and had unprotected sexual intercourse with 0–3 partners in the last 6 months prior to presentation. Men with CT infection were in median 29.4 years old (IQR: 19.8–30.0) and had unprotected sexual intercourse with 0–3 partners in the last 6 months prior to presentation. Syphilis was exclusively diagnosed in MSM. Of all clients, 45.6% (95% CI: 39.4–51.8) had protective anti-HBs titers.

MSM not taking PrEP diagnosed with at least one STI had a median age of 28.7 years (IQR: 27.3–31.7) and had unprotected sexual intercourse with 0–5 partners in the last 6 months prior to presentation. Among all MSM not taking PrEP, 65.8% (95% CI: 50.0–81.6) had protective antibody levels against HAV and 47.4% (95% CI: 30.7–64.0) had protective antibody levels against HBV.

MSM taking PrEP were older (median 35.0 years [range: 18.0–56.9] vs. 28.0 years [range: 21.5–73.1], *p* < 0.01), had higher educational attainment (university degree 49.4 vs. 34.0%, *p* < 0.01) and more condomless anal and vaginal sex than MSM not taking PrEP (*p* = 0.05). Total follow-up of PrEP users was 57.3 person-years and 25 STIs were diagnosed in 18 clients (22.2%, 95% CI: 13.0–31.5) resulting in 0.44 STI per person-year. During the study period, 20 (24.7%) individuals started PrEP and underwent regular HIV/STI screening, in which 1 of 7 (14.3%) syphilis cases, 4 of 10 (40.0%) CT infections, 1 of 6 (16.7%) NG infections, and 1 of 1 (100%) HIV infection were diagnosed. Thirty-five PrEP users (43.2%) received at least one vaccination against HAV and/or HBV during the study period, thus about 90% of PrEP users had protective antibody levels against HAV (*n* = 74, 91.4%, 95% CI: 83.0–96.5) and HBV (*n* = 72, 88.9%, 95 CI: 80.0–94.8) by the end of the study period. Thirty PrEP users (37.0%) resided outside of the district of Freiburg (Figure 1).

Twenty-two (61.1%) of patients with STI diagnosis were treated on-site that include 15 of 18 (83.3%) PrEP users. In the group not using PrEP, 8 of 12 men (66.6%) and 3 of 6 women (50.0%) did not receive treatment on-site. Of 14 patients not receiving on-site treatment, 5 lived outside of the district of Freiburg.

## Discussion

The Center for Sexual Health Freiburg was established in February 2017 as a low threshold, cross-sectoral, and on-site integrated STI/HIV counseling and treatment service providing anonymous and low-cost comprehensive STI/HIV counseling and testing in an MSM friendly environment. The cooperative approach of a local non-governmental organization supporting people with HIV in conjunction with infectious diseases or HIV physicians enabled us to offer on-site psycho-social care, STI therapy, vaccination against Hepatitis A and B, and to provide PrEP-related diagnostics and drug supply.

The Center for Sexual Health Freiburg was readily accepted by broad segments of the population characterized by a sexual behavior with a higher possibility for STI transmission and a high prevalence of STI. Most of the clients at the CSHF were Freiburg city dwellers, however, a substantial proportion of clients came from all parts of the region and beyond.

Men having sex with men represented roughly one-third of presenting clients. The cross-sectoral organization of CSHF that includes a local non-governmental organization supporting people with HIV working with peer educators creating a stigma-free environment with a positive approach to sexuality may have been an important factor to promote acceptance of CSHF in the local MSM community. MSM had condomless sexual encounters with more partners than heterosexual men and women and were disproportionately affected by STI, which is in accordance with existing data (6, 8, 14). In our study, the prevalence of STI in MSM not using PrEP was lower as compared to other studies carried out in Germany (8, 21). This difference is partly explained by the study setting that offered screening for asymptomatic individuals, yet the geographical localization of CSHF outside of known STI hotspots in Germany may also contribute. Hotspots of syphilis incidence, which is a notifiable disease in Germany, are located in metropolitan areas, suggesting divergent sexual behaviors of the population according to their residence (3). In Germany as in many other countries, MSM are disproportionately vulnerable to STI. In 2018, 85.9% of syphilis cases and 66.7% of newly diagnosed HIV infections were related to MSM, making MSM an important target group in the efforts to prevent and reduce HIV and STI transmission (3, 7, 14). German guidelines recommend STI/HIV screening of sexually active MSM every 3–6 months (13).

The burden of asymptomatic STI was also substantial in the population of heterosexual men and women presenting at CSHF. Prevalence rates of asymptomatic infections with CT were comparable with infection rates of studies carried out in German STI testing services at local public health authorities and in a similar cross-sectoral German healthcare center in Bochum, although the median age especially of infected women was higher in our study (6, 22). The study in Bochum focusing on young individuals between 14 and 31 years showed a low prevalence of NG in women in accordance with the results of our analysis (22). NG infection rates in men were not presented according to sexual orientation, thus so far there are no data on asymptomatic NG infection in heterosexual men (22). If German guidelines that recommend screening for CT, NG, syphilis, and HIV only in heterosexual individuals with 10 or more sexual partners per year would have been applied in our study, most of the STI diagnosed in heterosexual men and women would have missed and would have continued to contribute to the reservoir of asymptomatic carriers outside of established high prevalence groups (13). This is also true for women with CT in our study, where screening is only recommended for women aged under 25 years–all but one woman diagnosed with CT at CSHF were older than 25 years. Thus, guidelines may have to be adapted. Guidelines from the Centers for Disease Control and Prevention (CDC), USA recommend the screening for CT and NG in all sexually active women under 25 years of age, in women with a new sex partner, more than one sex partner, a sex partner with concurrent partners, and in pregnant women and for CT in men in high prevalence clinical settings, e.g., in STI clinics (23).

Given the often asymptomatic presentation of STI/HIV, enhanced STI/HIV screening is a key component in national and international STI/HIV prevention strategies (14, 15). As costs for comprehensive STI/HIV testing, which may easily exceed 200€ per screening, are not covered by the German compulsory health insurance in asymptomatic individuals, the threshold for individuals to access STI/HIV screening is high. Affordable STI testing services are only available at local public health authorities and non-governmental organizations. Measures to improve accessibility to STI/HIV testing in the German healthcare system would certainly help to increase the uptake of STI/HIV testing and contribute to reduce STI/HIV infection rates in Germany.

Pre-exposure prophylaxis with oral tenofovir disoproxil fumarate and emtricitabine (TDF-FTC) was approved in 2016 in Germany after having demonstrated to be a highly effective tool in preventing HIV infection in high-risk MSM (24–28). Since the German compulsory health insurance's coverage of costs for PrEP and related diagnostics, PrEP has quickly gained popularity, especially in the group of MSM. Recently published results of a study estimated the number of PrEP users to be about 15,600–21,600 and the number of men with unmet PrEP needs of about 27,500–93,000 by the end of June 2020 in Germany. Interestingly, the latter study found an unequal regional distribution with a positive correlation between satisfied PrEP needs and the number of regional physicians qualified to deliver PrEP (29). Thus, access to local services providing PrEP may be an important factor to promote PrEP uptake in Germany. All clients presenting at CSHF received sexual health counseling that includes information on PrEP. The cross-sectoral structure of CSHF facilitated the transfer of clients identified as in need of PrEP into the medical setting for further evaluation and prescription of PrEP. Consequently, 20 MSM started PrEP during the study period resulting in a total of 81 PrEP users followed up at CSHF making PrEP services an important activity of CSHF. A substantial proportion of PrEP users came from locations outside of the city of Freiburg, suggesting an unmet need for PrEP services at their place of residence. Establishing services proposing PrEP on the level of local subcenters may improve access to PrEP helping to scale up PrEP coverage and curb the local HIV epidemic (29).

Men having sex with men using PrEP had a high incidence of STI in our study, which is in line with results of previous studies showing higher STI incidence in MSM with PrEP as compared to MSM without PrEP or increasing incidence of STI after starting PrEP (8, 30–32). However, when compared to Berlin and eight other metropolitan areas known to be STI hotspots in Germany, clients had condomless sex with a smaller number of partners and STI prevalence was lower in our study (8). Increased STI incidence in MSM with PrEP has been attributed to an increased vulnerability to STI in individuals with a self-identified need for HIV PrEP and increases in condomless anal sex after initiation of PrEP (33, 34). These findings highlight the importance to locate PrEP services in settings with abundant experience in dealing with people with a high risk for STI. The cross-sectoral approach enabled us to offer appropriate preventive, diagnostic, and curative measures for STI and to provide access to professional sexual health counseling. In this setting, preventive measures to avoid infections are of utmost importance. CSHF showed a strong uptake of HAV and HBV vaccinations, resulting in protective antibody levels in about 90% of the PrEP users.

Roughly one-third of patients with diagnosed STI did not receive treatment on-site with the risk of delayed treatment and further transmission of infections. Especially in the setting of CSHF, situated in a regional center with a large catchment area, where clients travel long distances, the introduction of rapid on-site diagnostics may be beneficial to permit prompt recognition and treatment of STI within the same consultation. Studies have demonstrated that using rapid on-site diagnostics in STI clinics is feasible, reduces time to notification, prevents STI transmissions, and thus is cost saving (22, 35).

Our study has several limitations. The study took place during the SARS-CoV-2 pandemic affecting many public and private aspects of the population that includes the closure of public places, gastronomy, cultural events, curfews, and reduction of private contacts. We assume that these measures affected many aspects of sexual life, most probably affected STI prevalence and clients' motivation to present for STI screening. Data from the ISHC in Bochum showed decreased numbers of sexual partners during the SARS-CoV-19 pandemic and reduced numbers of clients presenting for STI testing with increased rates of test positivity in periods with high SARS-CoV-2 incidence (36). Many PrEP users suspended PrEP during the lockdown (37). At CSHF, the number of clients was markedly reduced during the study period as compared to 829 clients during the equivalent pre-pandemic period from 1 May 2019 to 28 February 2020, which may reflect the restricted access due to the introduction of appointment-based consultations. Nevertheless, the demand for appointments remained high at CSHF during the pandemic and services were running at full capacity while respecting hygiene standards.

Though a standardized comprehensive STI testing package was proposed to all clients, uptake of the respective tests reached only 83.3–89.7%. Thus, undiagnosed infections cannot be excluded from affecting STI/HIV prevalence in this study. Not being tested showed no association with sex, sexual orientation, place of birth, or educational attainment.

For economic reasons, samples from different anatomical sites were pooled for CT/NG NAAT at CSHF. Therefore, the study was not able to analyze site-specific CT/NG prevalence.

Because the history of STI prior to initiation of PrEP was not collected, the effect of PrEP initiation on STI incidence could not be evaluated.

Finally, the study population was young and MSM were overrepresented. Though the results of the study are not representative of the general population of Germany, our study characterizes well the local population in need of low threshold sexual health services.

In conclusion, the cross-sectoral and on-site integrated structures of CSHF successfully addressed an intersectoral gap in the German healthcare system and the national STI/HIV prevention strategy. Low threshold access to comprehensive STI/HIV counseling and testing was used by a broad spectrum of the population at risk and, moreover, allowed to identify a significant number of otherwise probably undetected infections also in clients not fulfilling high prevalence profiles defined in German screening guidelines. The cross-sectoral collaboration allowed on-site guideline-based treatment of patients with STI thereby minimizing the risk of ongoing transmission. PrEP uptake could be enhanced by on-site counseling and initiation of PrEP in clients identified with PrEP needs. Establishing structures following the example of ISHCs may be an important contribution to curb the STI/HIV epidemic in Germany.

## Data Availability Statement

The original contributions presented in the study are included in the article/Supplementary Material, further inquiries can be directed to the corresponding author.

## Ethics Statement

The studies involving human participants were reviewed and approved by Institutional Review Board of the University Medical Center of the University of Freiburg. The patients/participants provided their written informed consent to participate in this study.

## Author Contributions

MM contributed to the conception and design of the work, to the acquisition, analysis and interpretation of the data, and drafted the manuscript. SU contributed to the acquisition of data and critically revised the manuscript. SZ, AF, and UH contributed to the acquisition of the data and critically revised the manuscript. WK contributed to the conception and design of the work and critically revised the manuscript. SR contributed to the conception and design of the work, the interpretation of data, and critically revised the manuscript. All gave final approval and agreed to be accountable for all aspects of work ensuring integrity and accuracy.

## Funding

This research received funding from the German Federal Ministry of Health (ZMVI1-2519AUK103) to SR and from the Ministry for Social Affairs and Integration Baden-Württemberg (AZ:51-5422.1-300/5), Germany, to CSHF.

## Conflict of Interest

The authors declare that the research was conducted in the absence of any commercial or financial relationships that could be construed as a potential conflict of interest.

## Publisher's Note

All claims expressed in this article are solely those of the authors and do not necessarily represent those of their affiliated organizations, or those of the publisher, the editors and the reviewers. Any product that may be evaluated in this article, or claim that may be made by its manufacturer, is not guaranteed or endorsed by the publisher.
